# Alcohol Consumption during COVID among Women with an Existing Alcohol-Use Disorder

**DOI:** 10.3390/ijerph18189460

**Published:** 2021-09-08

**Authors:** Jessica D. Hanson, Carolyn Noonan, Amy Harris, Kyra Oziel, Michelle Sarche, Richard F. MacLehose, Marcia O’Leary, Dedra Buchwald

**Affiliations:** 1 Department of Applied Human Sciences, University of Minnesota Duluth, Duluth, MN 55812, USA; harr2542@d.umn.edu; 2 Institute for Research and Education to Advance Community Health, Washington State University, Seattle, WA 98101, USA; carolyn.noonan@wsu.edu (C.N.); kyra.oziel@wsu.edu (K.O.); dedra.buchwald@wsu.edu (D.B.); 3 Centers for American Indian and Alaska Native Health, Colorado School of Public Health, University of Colorado Anschutz Medical Campus, Aurora, CO 80045, USA; michelle.sarche@cuanschutz.edu; 4 Division of Epidemiology and Community Health, University of Minnesota, Minneapolis, MN 55454, USA; macl0029@umn.edu; 5 Missouri Breaks Industries Research Inc., Eagle Butte, SD 57625, USA; marcia.oleary@mbiri.com

**Keywords:** COVID-19 pandemic, alcohol, indigenous communities

## Abstract

Prior to the pandemic, our research team implemented a randomized controlled trial of an intervention to reduce risk for alcohol-exposed pregnancy (AEP) in American Indian women. When active recruitment for the in-person trial was paused due to COVID, the research team moved to conducting follow-up surveys with participants who had completed the intervention to better understand changes to their alcohol use during the pandemic. We collected surveys from 62 American Indian women who had completed the Native CHOICES intervention. Baseline data collected pre-COVID included demographics and scores on the Alcohol Use Disorders Identification Test (AUDIT). Follow-up surveys conducted during the active pandemic period included a self-reported questionnaire about changes in drinking patterns. At pre-COVID baseline, all participants were engaged in heavy or binge drinking. At follow-up during COVID, 24.2% reported drinking more, and over half had at least one binge drinking episode. Approximately half reported reduced drinking. We found that risky drinking remained an issue during the pandemic for many American Indian women who had engaged in this behavior pre-COVID, while others reported reducing their alcohol consumption. As the pandemic abates, concerted efforts must be made to reach those with identified alcohol use disorders to offer resources and intervention as needed.

## 1. Background

The COVID-19 pandemic generated shutdowns across borders, which induced changes in behavior patterns in people around the world, including alcohol consumption in different subpopulations. Stay-at-home orders, unemployment, and a high death count all contribute to pandemic-related psychological stress [1,2]. Additionally, more relaxed alcohol laws permitting the curbside or home delivery of alcohol have contributed to a 54% increase in sales at liquor stores and a 262% increase in online/home delivery alcohol sales compared to sales data from the same week in 2019 [3]. A review of emergency department visits in a United States (U.S.) healthcare system found that the number of alcohol-related complaints increased from 28.2% to 33.5% [3]. Notably, risky drinking increased among those under lockdowns compared to those in places without such restrictions [4]. Increased drinking has been particularly pronounced among people with children, those with existing mental health concerns, and those less socially connected [5,6,7,8].

An increase in drinking during the pandemic is particularly seen in women. Pre-COVID, about 32% of females worldwide aged 15 and older consumed alcohol and drinking among women was on the increase [9]. For example, national surveys conducted between 2000 and 2016 found that the number of women aged 18 and older who drank alcohol increased by 6%, while the number of women who binge drank increased by 14% [9]. Preliminary data on drinking during the COVID-19 pandemic highlights that women may be at particular risk for increased and risky drinking [7]. A study of mainly female participants in the U.S. found that one-third (34%) reported binge drinking in the past month during the pandemic [3]. A separate follow-up study during the early stages of the pandemic and then several months after widespread COVID-19 restrictions were implemented found that the frequency of alcohol consumption among women increased overall by 0.78 days, or 17%, over baseline [10]. A smaller study primarily of women from two Midwestern states in the U.S. found that Alcohol Use Disorders Identification Test (AUDIT) scores increased significantly among women during the pandemic and that new cases of alcohol abuse were mostly among women [8]. In general, increases in both the frequency and quantity of alcohol consumed were observed in the sample and, in particular, increases in the frequency of alcohol consumed prior to 5:00 p.m. [8].

Compared to other racial/ethnic groups, American Indian/Alaska Native (AI/AN) women are more likely to abstain from alcohol, but among those who do drink, bingeing patterns of alcohol consumption are more likely [11,12]. AI/AN women are also more likely to report drinking alcohol during pregnancy (16.2%) [13], in contrast to women sampled nationally (10.2%) [14]. However, it is unknown how the COVID-19 pandemic impacted these drinking patterns. The focus of this study, therefore, is to evaluate alcohol consumption during the pandemic among AI/AN women who exhibited risky drinking patterns prior to the pandemic.

## 2. Methods

### 2.1. Recruitment and Eligibility

Follow-up was conducted with AI/AN women from a single tribe who previously enrolled in a randomized controlled trial (RCT) of Native CHOICES (Changing High-risk Alcohol Use and Increasing Contraception Effectiveness Study), an in-person alcohol-exposed pregnancy (AEP) prevention program [15]. RCT inclusion criteria included: age (18–44 years old); drinking patterns (self-reported binge, 4+ drinks/episode; or heavy drinking, 8+ drinks/week); pregnancy status (not currently pregnant); and ability to get pregnant (biologically able to become pregnant, sexually active, and not using contraceptives at all or effectively). The Native CHOICES intervention consisted of two in-person motivational interviewing sessions, one elective contraceptive counseling session, and optional supportive electronic messaging. There were four major data collection points: baseline, and six-weeks, three-months, and six-months post-baseline. The study was conducted under the review of the Great Plains IRB, the Washington State University IRB, and with approvals from the tribe involved with Native CHOICES.

Due to the COVID-19 pandemic, several adjustments were made to the Native CHOICES study [15], including the addition of a COVID-19 impact supplemental survey. The COVID-19 impact survey was developed to try to account for the behavioral impacts of COVID-19 on participants that related specifically to AEP risk (e.g., alcohol consumption and contraceptive use). We received additional approvals from each of the entities above to collect COVID impact follow-up data from Native CHOICES participants. All individuals who were enrolled in the Native CHOICES study in either the intervention or control arm from approximately 1 March 2019 (when active recruitment began), to 12 March 2020 (immediately prior to the COVID-19 pandemic and subsequent lockdowns), were eligible to be re-contacted for this supplemental study. These participants were called via telephone to determine their interest in answering additional COVID-19 related questions. Participants who agreed were re-consented over the telephone using an abbreviated verbal consent. Data collection was completed immediately after consent was acquired, and participants received $10 for taking part. 

### 2.2. Measures and Data Analysis

Relevant pre-pandemic baseline study data included sociodemographic characteristics and the results of the AUDIT screening tool. The total AUDIT score, which ranged from 0 to 40 with higher scores indicative of potentially hazardous or harmful alcohol consumption, was calculated as the sum of all 10 AUDIT items. 

Drinking behavior during the pandemic was assessed by the following four questions: (1) Compared to before the COVID-19 pandemic, would you say you are drinking more, about the same, or less? (2) Since the COVID-19 pandemic began, has there been a time that you had four or more standard drinks in a single day? (3) About how many days did you have four or more standard drinks in a single day? and (4) Since the COVID-19 pandemic began, has there ever been a time that you had 8 or more standard drinks in 1 week? 

To analyze data, means and standard deviations were used for continuous variables, and percentages were used for categorical variables to describe participant characteristics and alcohol consumption. Descriptive statistics for sociodemographic characteristics were calculated separately for eligible participants who did and did not complete the COVID-19 supplemental questionnaire. We also calculated 95% confidence intervals for estimates of alcohol consumption during the pandemic and sociodemographic characteristics according to consumption during the pandemic. We did not use formal statistical testing due to the modest sample size and the exploratory nature of the analysis. Results of questions on contraceptive use and access during the pandemic are not included here as the focus is on alcohol consumption results.

## 3. Results

There were 122 participants who were eligible for the follow-up COVID impact survey. Of these, 62 consented to completing our questionnaire on alcohol consumption during the pandemic. Most baseline sociodemographic characteristics were similar in participants who did and did not complete the questionnaire, although participants who completed the questionnaire may have more often had a permanent residence and experienced less homelessness than participants who did not complete the questionnaire (Table 1). Average AUDIT scores were slightly higher in participants who completed versus did not complete the survey (18.2 ± 8.4 and 16.2 ± 8.9, respectively), but additional comparisons were not completed because all participants were drinking at a risky level at baseline, as it was part of the eligibility for the program. COVID impact data were collected between October 2020 and January 2021, or approximately 8–11 months after the pandemic and official lockdowns began in March 2020. The time difference between when baseline data and COVID impact survey data were collected ranged from 8 to 20 months, with an average of 15 months.

The average age of participants who completed the COVID impact survey was 31.4 years old (±6.7). Based on Native CHOICES eligibility criteria, all survey respondents were known to be at-risk for a pregnancy (e.g., sexually active with a male; able to get pregnant; not using effective or any contraception) at the pre-COVID baseline. At baseline (e.g., pre-COVID), participants’ AUDIT score corresponded to the moderate-severe AUD level with an average of 18.2 (±8.4), and range of 5 to 35. Table 1 provides additional sociodemographic details of the participants, including education, employment status, household income, and housing status. 

The COVID impact survey data showed (Table 2) that n = 15 (24.2%) of participants felt they were consuming more alcohol during the pandemic than before COVID-19 emerged; n = 14 (22.6%) reported drinking about the same amount as before COVID-19; and n = 33 (53.2%) reported drinking less during the pandemic (Table 2). A total of n = 31 (52.5%) stated they had at least one bingeing episode (e.g., 4 or more standard drinks in a single day) since the pandemic began. Among participants, the average number of days with at least one binge episode (since the pandemic began) was 2.9 (95% CI: 2.3, 3.6), with a range 1 to 7. Of the participants who had at least one bingeing episode during the pandemic, n = 34 (54.8%) also had at least one additional heavy drinking episode or 8+ drinks in a week. 

A comparison of select sociodemographic characteristics according to alcohol consumption during the pandemic showed that participants who reported drinking less during compared to before the pandemic may have been less often married or had a significant other and more often had children sleeping in their residence than participants who reported drinking the same or more during the pandemic (Table 3). Results should be interpreted with caution due to imprecise estimates.

## 4. Discussion

While some hypothesized that alcohol use would decrease during the pandemic due to reduced alcohol availability and diminished financial resources [6], others hypothesized that “COVID-19 self-isolation periods may lead to an increased risk of alcohol relapse, misuse, and the development of alcohol use disorders in at-risk individuals” [16]. The goal of this study was to determine whether there were changes in alcohol consumption during the COVID-19 pandemic among AI/AN women who were drinking at risky levels prior to the pandemic. 

Among this cohort of women, approximately half reported a decrease in their alcohol consumption during the pandemic while approximately a quarter reported an increase and a quarter stated they did not change their consumption. While self-reported alcohol consumption appeared to decrease during the pandemic for many, there were still incidents of binge and heavy drinking during the pandemic. In addition, participants who reported drinking less during compared to before the pandemic were less often married or had a significant other and more often had children sleeping in the home. While these results should be interpreted carefully due to the limitations of this study, this is disparate from other studies that show that drinking increased during the pandemic in households with children, higher stress levels, and reduced social connectedness [5,6,7,8].

In all, it appeared that this group of AI/AN women experienced a very heterogeneous response to the pandemic with respect to alcohol consumption. It is unclear why some of the AI/AN women who were drinking at binge levels prior to the pandemic continued or intensified their drinking patterns during the pandemic, while others reported drinking less than before the pandemic. It is possible that many AI/AN women, accustomed to responding to trauma or stressful situations or because of their previous involvement in the AEP prevention program, were able to persevere and had the resilience and protective factors necessary to respond to the pandemic. It is also possible that due to enrollment in an AEP prevention program, participants were providing more socially acceptable responses to the follow-up survey. 

What is known, however, is that this population faced unique pandemic-related stressors, such as higher per capita rates of COVID-19. According to Hatcher et al. (2020), “the cumulative incidence of laboratory-confirmed COVID-19 among AI/AN persons was 3.5 times that among non-Hispanic white persons.” Pre-existing social conditions, such as crowded living conditions, lack of running water, under-resourced healthcare delivery by the U.S. federal government, and ongoing health disparities in the population made many AI/AN communities particularly vulnerable to the most severe impacts of the virus [17,18]. Because of this, many AI/AN communities maintained stricter stay-at-home policies than many non-tribal communities, and it is unsurprising that many participants in the study continued drinking at high levels and in some cases increased their drinking. 

Based on the results of this study, several public health considerations must be taken into account. First, the cohort described here was enrolled in a study to reduce risky drinking, but the pandemic may have subsequently altered risky drinking trajectories, generating the need for the sample to ‘reset’ their goals around improving alcohol behaviors. However, there are few to no other resources to reduce risky drinking in this resource-poor community, whose members will be healing from the cultural and familial impacts of the pandemic for years to come. Of additional concern is that the present sample enrolled in Native CHOICES because they were at risk for an AEP; the goal of Native CHOICES is to reduce the likelihood of children born with fetal alcohol syndrome or other fetal alcohol spectrum disorders. In an editorial, Sher (2020) discusses the “collateral damage” caused by COVID-19 and expresses the concern that the heightened risk related to alcohol consumption (e.g., FASD and AEP) has not been addressed. As noted in the editorial, “Although we might soon enter a post-COVID era, new cases of FASD will persist for decades and permanently compromise the lives and life chances of those affected” [19].

As we emerge from the pandemic, there may be long-term impacts of these pandemic-related behavioral and mental health issues [1], and there is likely to be “prolonged adverse psychosocial, interpersonal, occupational, and health impacts as the world attempts to recover from the pandemic crisis” [4]. Clay and Parker (2020) conclude that the increase in risk for alcohol relapse and misuse will almost certainly cause strain on addiction services and health services during and after the pandemic [16]. This is of particular concern for many tribal communities that already lack adequate treatment resources, particularly culturally congruent services. Challenges in accessing any type of alcohol treatment both during and after the pandemic will only aggravate and possibly increase alcohol use disorders [20]—something that must be addressed in our post-COVID world. 

There are some limitations to the present study, and the results should be interpreted carefully. The sample was small, from a single rural community, and the group was homogenous with respect to existing pre-pandemic risky drinking patterns and other demographic features. There was relatively low response to the supplemental COVID impact follow-up survey, so we lack data on the drinking patterns of those who did not respond or whom we were unable to reach. We did not include a comparison group or control group of AI/AN women who had not exhibited pre-pandemic risky drinking patterns. As with other, similar studies of drinking during COVID, this is a descriptive study with no follow-up. Additional studies are warranted to evaluate the impact of the COVID-19 pandemic on drinking patterns. Further, the present study was conducted within the context of an ongoing randomized trial; if the intervention is effective in decreasing drinking as is hypothesized, some of the increased drinking reported here could be due to a rebound effect after the end of the intervention rather than the pandemic. 

## 5. Conclusions

With job losses disproportionately impacting women [21] and with many women playing multiple, conflicting roles of at-home worker, primary caregiver, and homeschooling teacher [22], it is not surprising that the risks for increased drinking delineated by others—households with children, higher stress levels, and reduced social connectedness [5,6,7,8]—are impacting women at higher levels across the U.S. Our study, however, showed that some AI/AN women may have had the resilience to overcome many of the pandemic-related stressors, either because of their involvement in an AEP prevention program pre-pandemic, because of abilities built in because of past stressors or traumas, or because of response bias. Regardless, the results contribute to existing research on drinking during the pandemic and highlight the necessity to begin immediately addressing the need for substance use treatment. Sugarman and Greenfield (2021) suggest that public health messaging, early intervention, and access to treatment are only a few ways to help individuals whose alcohol consumption has increased during the COVID-19 pandemic [23]. These interventions should be made accessible to varying populations via telehealth and other virtual platforms and should address potential co-occurring medical conditions that result from increased and excessive drinking [23]. 

## Figures and Tables

**Table 1:** Baseline sociodemographic characteristics for American Indian participants of the NCARE CHOICES study who were eligible to complete a COVID-19 supplemental questionnaire, according to completion status.

Characteristic	Completed Questionnaire (N = 62) %	Eligible but Did Not Complete Questionnaire (N = 60) %
Age, mean years (SD)	31.4 (6.7)	32.4 (7.3)
Completed education		
<High school	33.9	37.3
High school or GED	37.1	33.9
Some college or more	29.0	28.8
Employed full- or part-time	19.4	10.0
Household income, %		
<$5000/year—$415/month or less	45.2	50.0
≥$5000/year—$416+/month	25.8	20.0
Do not want to answer	29.0	30.0
Receive public assistance in past 12 months	85.5	86.7
Have a permanent residence (house, apartment, trailer)	61.3	45.0
Experienced homelessness in past 6 months	29.0	43.3
Married or significant other	46.8	45.0
Number of adults slept in your residence last night, including self		
1	14.5	11.7
2	30.7	25.0
3	19.4	23.3
4 or more	35.5	40.0
Number of children (age 0–17 years) slept in your residence last night		
0	27.4	30.5
1–2	32.3	32.2
3–4	24.2	25.4
5 or more	16.1	11.9

Note: % presented unless otherwise noted; NCARE CHOICES = Native Center for Alcohol Research and Education—Changing High-risk Alcohol Use and Increasing Contraception Effectiveness Study; SD = standard deviation; GED = General Education Diploma.

**Table 2:** Drinking behaviors during the pandemic in American Indian participants of the NCARE CHOICES study who completed a COVID-19 supplemental questionnaire.

Drinking Behavior	% (95% CI)
Among all participants:	(N = 62)
Alcohol consumption during pandemic compared to before, *%*	
More now than before the pandemic	24.2 (14.2, 36.7)
About the same amount as before the pandemic	22.6 (12.9, 35.0)
Less now than before pandemic	53.2 (40.1, 66.0)
Had 4 or more standard drinks in a single day, since pandemic began	50.0 (37.0, 63.0)
Among participant who had 4 or more drinks in a single day:	(N = 31)
# of days with 4+ standard drinks in a single day, *mean days (95% CI)*	2.9 (2.3, 3.6)
Had 8 or more standard drinks in a week since pandemic began, *%*	54.8 (36.0, 72.7)

NCARE CHOICES = Native Center for Alcohol Research and Education—Changing High-risk Alcohol Use and Increasing Contraception Effectiveness Study; CI = confidence interval.

**Table 3:** Select baseline sociodemographic characteristics for American Indian participants of the NCARE CHOICES study who completed a COVID-19 supplemental questionnaire, according to drinking behavior during the pandemic.

	Alcohol Consumption during Compared to before Pandemic		
Characteristic	More Now (N = 15) % (95% CI)	Same (N = 14) % (95% CI)	Less Now (N = 33) % (95% CI)
Age, mean years (95% CI)	34.1 (30.0, 38.1)	30.2 (26.4, 34.0)	30.7 (28.5, 33.0)
Completed education			
<High school	26.7 (7.8, 55.1)	42.9 (17.7, 71.1)	33.3 (18.0, 51.8)
High school or GED	40.0 (16.3, 67.7)	21.4 (4.7, 50.8)	42.4 (25.5, 60.8)
Some college or more	33.3 (11.8, 61.6)	35.7 (12.8, 64.9)	24.2 (11.1, 42.3)
Employed full- or part-time	26.7 (7.8, 55.1)	14.3 (1.8, 42.8)	18.2 (7.0, 35.5)
Household income			
<$5000/year—$415/month or less	26.7 (7.8, 55.1)	64.3 (35.1, 87.2)	45.5 (28.1, 63.6)
≥$5000/year—$416+/month	33.3 (11.8, 61.6)	28.6 (8.4, 58.1)	21.2 (9.0, 38.9)
Do not want to answer	40.0 (16.3, 67.7)	7.1 (0.2, 33.9)	33.3 (18.0, 51.8)
Married or significant other	46.7 (21.3, 73.4)	71.4 (41.9, 91.6)	36.4 (20.4, 54.9)
≥1 child (age 0–17 years) slept in your residence last night	53.3 (26.6, 78.7)	71.4 (41.9, 91.6)	81.8 (64.5, 93.0)

Note: % presented unless otherwise noted; NCARE CHOICES = Native Center for Alcohol Research and Education—Changing High-risk Alcohol Use and Increasing Contraception Effectiveness Study; CI = confidence interval; GED = General Education Diploma.

## Data Availability

The data are not publicly available due to tribal sovereignty; data are owned and controlled by the tribal partner involved with this project and can only be accessed with approval from the tribal council.
